# Methyl 4-hy­droxy-1,1-dioxo-2-(2-phenyl­eth­yl)-2*H*-1λ^6^,2-benzothia­zine-3-carboxyl­ate

**DOI:** 10.1107/S160053681104966X

**Published:** 2011-11-25

**Authors:** Muhammad Nadeem Arshad, Islam Ullah Khan, Muhammad Zia-ur-Rehman, Muhammad Danish, K. Travis Holman

**Affiliations:** a Department of Chemistry, University of Gujrat (H. H. Campus), Gujrat 50700, Pakistan; b Materials Chemistry Laboratory, Department of Chemistry, GC University, Lahore 54000, Pakistan; c Applied Chemistry Research Centre PCSIR Laboratories Complex, Lahore 54600, Pakistan; d Department of Chemistry, Georgetown University, 37th and O St NW, Washington, DC 20057, USA

## Abstract

In the title compound, C_18_H_17_NO_5_S, the thia­zine ring adopts a half-chair conformation and the dihedral angle between the aromatic rings is 79.41 (6)°. An intra­molecular O—H⋯O hydrogen bond generates an *S*(6) ring. In the crystal, mol­ecules are linked by weak C—H⋯O inter­actions resulting in infinite sheets along the *b* and *c* axes.

## Related literature

For related structures, see: Arshad *et al.* (2011*a* ▶); Ahmad *et al.* (2010 ▶); Khalid *et al.* (2010 ▶). For further synthetic details, see: Arshad *et al.* (2011*b* ▶). For graph-set notation, see: Bernstein, *et al.* (1995 ▶). For ring conformations, see: Cremer & Pople (1975 ▶).
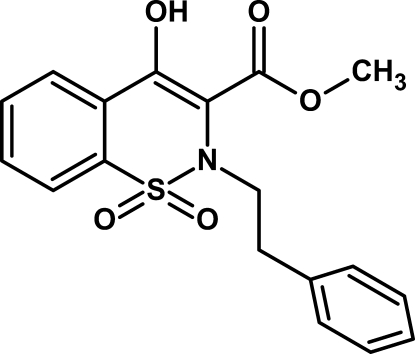


## Experimental

### 

#### Crystal data

C_18_H_17_NO_5_S
*M* *_r_* = 359.39
Monoclinic, 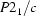

*a* = 9.018 (2) Å
*b* = 19.026 (4) Å
*c* = 10.193 (2) Å
β = 106.441 (3)°
*V* = 1677.3 (7) Å^3^
*Z* = 4
Mo *K*α radiation
μ = 0.22 mm^−1^
*T* = 100 K
0.37 × 0.23 × 0.08 mm

#### Data collection

Bruker SMART 1K diffractometer with a Bruker APEXII CCD detector
Absorption correction: multi-scan (*SADABS*; Bruker, 2007 ▶) *T*_min_ = 0.922, *T*_max_ = 0.982
19773 measured reflections
4066 independent reflections
2735 reflections with *I* > 2σ(*I*)
*R*_int_ = 0.094

#### Refinement

*R*[*F* ^2^ > 2σ(*F* ^2^)] = 0.049
*wR*(*F* ^2^) = 0.116
*S* = 1.01
4066 reflections
230 parameters
H atoms treated by a mixture of independent and constrained refinement
Δρ_max_ = 0.38 e Å^−3^
Δρ_min_ = −0.44 e Å^−3^

### 

Data collection: *SMART* (Bruker, 2001 ▶); cell refinement: *SAINT* (Bruker, 2001 ▶); data reduction: *SAINT*; program(s) used to solve structure: *SHELXS97* (Sheldrick, 2008 ▶); program(s) used to refine structure: *SHELXL97* (Sheldrick, 2008 ▶); molecular graphics: *PLATON* (Spek, 2009 ▶); software used to prepare material for publication: *X-SEED* (Barbour, 2001 ▶), *WinGX* (Farrugia, 1999 ▶) and *PLATON*.

## Supplementary Material

Crystal structure: contains datablock(s) I, global. DOI: 10.1107/S160053681104966X/hb6520sup1.cif

Structure factors: contains datablock(s) I. DOI: 10.1107/S160053681104966X/hb6520Isup2.hkl

Supplementary material file. DOI: 10.1107/S160053681104966X/hb6520Isup3.cml

Additional supplementary materials:  crystallographic information; 3D view; checkCIF report

## Figures and Tables

**Table 1:** Hydrogen-bond geometry (Å, °)

*D*—H⋯*A*	*D*—H	H⋯*A*	*D*⋯*A*	*D*—H⋯*A*
C2—H2⋯O3^i^	0.95	2.55	3.391 (3)	147
C17—H17⋯O2^ii^	0.95	2.53	3.205 (3)	128
C18—H18⋯O4^iii^	0.95	2.49	3.308 (3)	145
O1—H1⋯O4	0.92 (3)	1.74 (3)	2.583 (2)	152 (3)

Symmetry codes: (i) 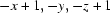; (ii) 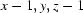; (iii) 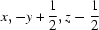.

**Fig. 1.**
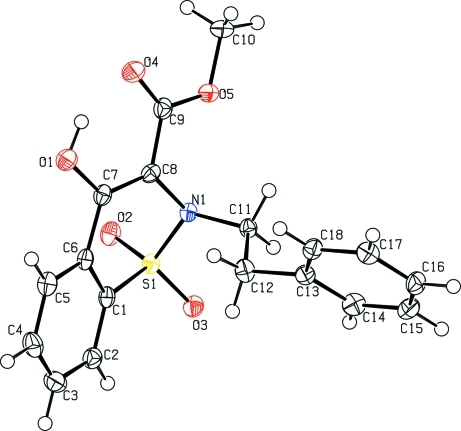
The labeled ortep diagram of (I) for which thermal ellipsoids were drawn at 50% probability level.

**Fig. 2.**
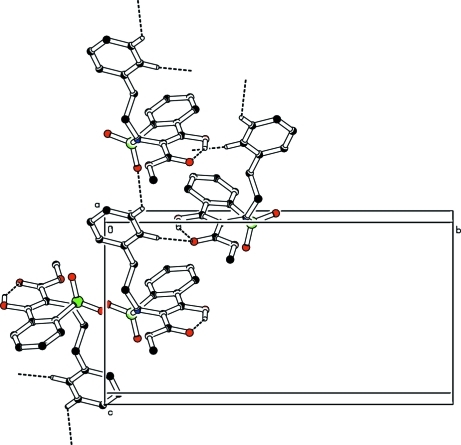
Unit cell packing for (I) showing the hydrogen bondings using dashed lines.

## supplementary crystallographic information

### Comment

Benzothiazines molecules are well explored for their crystallographic studies (Arshad *et al.* 2011*a*), (Ahmad *et al.* 2010), (Khalid *et al.* 2010).

In the title compound, the nitrogen atom of methyl-4-hydroxy-2*H*-1, 2-benzothiazine-3-carboxylate 1,1-dioxide was alkylated with phenylethyl group. The thiazine ring (with root mean square deviation = 0.210Å) is oriented at dihedral angle of 13.12 (11)° with respect to the aromatic ring (C1—C6) and the S1 & N1 atoms showed deviations from the least square plane by 0.3121 (10)Å and -0.3310 (12)Å, respectively. The thiazine ring adopts a half chair conformation with puckering amplitude Q = 0.5160 (16)Å θ = 63.6 (2)°, φ = 26.3 (2)° (Cremer & Pople, 1975). The phenyl ring (C13—C17) is oriented at dihedral angle of 79.41 (6)° and 66.53 (5)° with  respect to the aromatic (C1—C6) and thiazine rings respectively. The intramolecular O–H···O interaction observed and generates an almost planer S(6) ring (Bernstein, *et al.*, 1995) with the r.m.s deviaton of 0.0131Å and produces dihedral angles of 16.02 (33)° & 15.87 (32)° with respect to the thiazine and aromatic (C1—C6) rings respectively.

Weak hydrogen bonding interactions of C—H···O type connect the molecules. The interaction C2—H2···O3 resulted in the formation of dimers which are further linked along *b* and  *c* axes (Fig.2. Tab.1).

### Experimental

The title compound was synthesised according to literature procedure (Arshad *et al.* 2011*b*) and crystalized in methanol under slow evaporation to yield colourless blocks.

### Refinement

All the C—H H-atoms were positioned with idealized geometry with C—H*_aromatic_* = 0.95 Å, C—H*_methylene_* = 0.99 Å and C—H*_methyl_* = 0.98 Å and were refined using a riding model with *U*_iso_(H) = 1.2 *U*_eq_for aromatic C atoms. The O—H H-atom was located via fourier map with O—H = 0.92 (3) Å with *U*_iso_(H) = 1.5 *U*_eq_for O atom.

### Crystal data

**Table:**

C_18_H_17_NO_5_S	*F*(000) = 752
*M**_r_* = 359.39	*D*_x_ = 1.423 Mg m^−^^3^
Monoclinic, *P*2_1_/*c*	Mo *K*α radiation, λ = 0.71073 Å
Hall symbol: -P 2ybc	Cell parameters from 2258 reflections
*a* = 9.018 (2) Å	θ = 2.3–22.9°
*b* = 19.026 (4) Å	µ = 0.22 mm^−^^1^
*c* = 10.193 (2) Å	*T* = 100 K
β = 106.441 (3)°	Block, colorless
*V* = 1677.3 (7) Å^3^	0.37 × 0.23 × 0.08 mm
*Z* = 4	

### Data collection

**Table:**

Bruker SMART 1K diffractometer with a Bruker APEXII CCD detector	4066 independent reflections
Radiation source: fine-focus sealed tube	2735 reflections with *I* > 2σ(*I*)
graphite	*R*_int_ = 0.094
φ and ω scans	θ_max_ = 28.4°, θ_min_ = 2.1°
Absorption correction: multi-scan (*SADABS*; Bruker, 2007)	*h* = −11→11
*T*_min_ = 0.922, *T*_max_ = 0.982	*k* = −25→25
19773 measured reflections	*l* = −13→13

### Refinement

**Table:**

Refinement on *F*^2^	Primary atom site location: structure-invariant direct methods
Least-squares matrix: full	Secondary atom site location: difference Fourier map
*R*[*F*^2^ > 2σ(*F*^2^)] = 0.049	Hydrogen site location: inferred from neighbouring sites
*wR*(*F*^2^) = 0.116	H atoms treated by a mixture of independent and constrained refinement
*S* = 1.01	*w* = 1/[σ^2^(*F*_o_^2^) + (0.0449*P*)^2^ + 0.2677*P*] where *P* = (*F*_o_^2^ + 2*F*_c_^2^)/3
4066 reflections	(Δ/σ)_max_ < 0.001
230 parameters	Δρ_max_ = 0.38 e Å^−^^3^
0 restraints	Δρ_min_ = −0.44 e Å^−^^3^

### Special details

**Table:**

Geometry. All s.u.'s (except the s.u. in the dihedral angle between two l.s. planes) are estimated using the full covariance matrix. The cell s.u.'s are taken into account individually in the estimation of s.u.'s in distances, angles and torsion angles; correlations between s.u.'s in cell parameters are only used when they are defined by crystal symmetry. An approximate (isotropic) treatment of cell s.u.'s is used for estimating s.u.'s involving l.s. planes.
Refinement. Refinement of F^2^ against ALL reflections. The weighted R-factor wR and goodness of fit S are based on F^2^, conventional R-factors R are based on F, with F set to zero for negative F^2^. The threshold expression of F^2^ > 2σ(F^2^) is used only for calculating R-factors(gt) etc. and is not relevant to the choice of reflections for refinement. R-factors based on F^2^ are statistically about twice as large as those based on F, and R- factors based on ALL data will be even larger.

### Fractional atomic coordinates and isotropic or equivalent isotropic displacement parameters (Å^2^)

**Table:**

	*x*	*y*	*z*	*U*_iso_*/*U*_eq_	
S1	0.29319 (6)	0.07784 (3)	0.51969 (5)	0.01673 (14)	
O3	0.30860 (16)	0.00885 (8)	0.46983 (15)	0.0214 (4)	
O5	−0.10038 (17)	0.12689 (8)	0.61803 (15)	0.0231 (4)	
O1	0.13070 (18)	0.28877 (8)	0.48807 (17)	0.0251 (4)	
O4	−0.07394 (18)	0.24350 (9)	0.60086 (17)	0.0276 (4)	
N1	0.10892 (18)	0.09548 (9)	0.48635 (17)	0.0154 (4)	
O2	0.36535 (16)	0.09408 (8)	0.65995 (14)	0.0227 (4)	
C1	0.3551 (2)	0.14133 (12)	0.4212 (2)	0.0170 (5)	
C7	0.1578 (2)	0.22064 (12)	0.4703 (2)	0.0182 (5)	
C8	0.0779 (2)	0.16750 (11)	0.5085 (2)	0.0177 (5)	
C5	0.3377 (2)	0.26007 (12)	0.3379 (2)	0.0220 (5)	
H5	0.2933	0.3057	0.3300	0.026*	
C9	−0.0383 (2)	0.18329 (12)	0.5789 (2)	0.0207 (5)	
C6	0.2849 (2)	0.20757 (11)	0.4087 (2)	0.0180 (5)	
C2	0.4731 (2)	0.12640 (12)	0.3629 (2)	0.0214 (5)	
H2	0.5193	0.0811	0.3720	0.026*	
C17	−0.3703 (2)	0.07609 (13)	−0.0561 (2)	0.0235 (5)	
H17	−0.4494	0.1074	−0.1035	0.028*	
C18	−0.2463 (2)	0.10137 (12)	0.0471 (2)	0.0209 (5)	
H18	−0.2404	0.1500	0.0694	0.025*	
C11	−0.0022 (2)	0.05922 (11)	0.3704 (2)	0.0170 (5)	
H11A	−0.1082	0.0670	0.3773	0.020*	
H11B	0.0185	0.0081	0.3786	0.020*	
C3	0.5220 (3)	0.17932 (13)	0.2911 (2)	0.0270 (6)	
H3	0.6021	0.1701	0.2496	0.032*	
C15	−0.2653 (3)	−0.03981 (13)	−0.0196 (2)	0.0247 (5)	
H15	−0.2717	−0.0884	−0.0421	0.030*	
C4	0.4549 (3)	0.24549 (13)	0.2793 (2)	0.0257 (5)	
H4	0.4901	0.2813	0.2303	0.031*	
C12	0.0052 (2)	0.08358 (12)	0.2302 (2)	0.0225 (5)	
H12A	0.0047	0.1356	0.2273	0.027*	
H12B	0.1029	0.0671	0.2146	0.027*	
C14	−0.1420 (3)	−0.01493 (13)	0.0835 (2)	0.0249 (5)	
H14	−0.0642	−0.0467	0.1314	0.030*	
C13	−0.1300 (2)	0.05577 (12)	0.1181 (2)	0.0196 (5)	
C16	−0.3799 (3)	0.00572 (13)	−0.0903 (2)	0.0239 (5)	
H16	−0.4643	−0.0113	−0.1619	0.029*	
C10	−0.2150 (3)	0.13972 (14)	0.6903 (3)	0.0314 (6)	
H10A	−0.2992	0.1682	0.6329	0.047*	
H10B	−0.1669	0.1649	0.7756	0.047*	
H10C	−0.2564	0.0948	0.7111	0.047*	
H1	0.057 (3)	0.2878 (14)	0.534 (3)	0.047*	

### Atomic displacement parameters (Å^2^)

**Table:**

	*U*^11^	*U*^22^	*U*^33^	*U*^12^	*U*^13^	*U*^23^
S1	0.0166 (2)	0.0155 (3)	0.0161 (3)	0.0009 (2)	0.0015 (2)	0.0007 (2)
O3	0.0204 (7)	0.0153 (8)	0.0269 (8)	0.0031 (6)	0.0042 (7)	−0.0005 (7)
O5	0.0245 (8)	0.0217 (9)	0.0271 (9)	−0.0026 (7)	0.0138 (7)	−0.0042 (7)
O1	0.0285 (9)	0.0147 (9)	0.0347 (10)	0.0009 (7)	0.0130 (8)	−0.0018 (7)
O4	0.0287 (9)	0.0193 (9)	0.0370 (10)	0.0027 (7)	0.0127 (8)	−0.0047 (7)
N1	0.0155 (8)	0.0138 (10)	0.0158 (9)	0.0005 (7)	0.0025 (7)	0.0009 (7)
O2	0.0237 (8)	0.0235 (9)	0.0171 (8)	−0.0012 (7)	−0.0005 (7)	0.0002 (7)
C1	0.0154 (9)	0.0186 (12)	0.0136 (10)	−0.0025 (9)	−0.0016 (8)	−0.0005 (9)
C7	0.0188 (10)	0.0161 (12)	0.0172 (11)	0.0004 (9)	0.0009 (9)	−0.0006 (9)
C8	0.0190 (10)	0.0174 (12)	0.0152 (10)	0.0025 (9)	0.0026 (9)	−0.0016 (9)
C5	0.0237 (11)	0.0182 (12)	0.0211 (12)	−0.0006 (9)	0.0018 (10)	0.0023 (9)
C9	0.0182 (10)	0.0210 (13)	0.0204 (11)	−0.0007 (9)	0.0015 (9)	−0.0017 (10)
C6	0.0175 (10)	0.0179 (12)	0.0158 (11)	−0.0035 (9)	0.0002 (9)	−0.0018 (9)
C2	0.0190 (10)	0.0219 (13)	0.0209 (11)	−0.0003 (9)	0.0020 (9)	−0.0029 (9)
C17	0.0217 (11)	0.0287 (14)	0.0186 (11)	0.0019 (10)	0.0035 (9)	0.0039 (10)
C18	0.0259 (11)	0.0193 (12)	0.0177 (11)	−0.0029 (10)	0.0067 (10)	−0.0004 (9)
C11	0.0162 (10)	0.0177 (12)	0.0160 (10)	−0.0016 (8)	0.0030 (9)	−0.0007 (8)
C3	0.0223 (11)	0.0353 (16)	0.0248 (12)	−0.0029 (10)	0.0089 (10)	−0.0019 (11)
C15	0.0301 (12)	0.0205 (13)	0.0243 (12)	0.0002 (10)	0.0089 (10)	−0.0030 (10)
C4	0.0256 (12)	0.0267 (14)	0.0245 (13)	−0.0067 (10)	0.0066 (10)	0.0046 (10)
C12	0.0227 (10)	0.0248 (13)	0.0182 (11)	−0.0036 (10)	0.0030 (9)	0.0031 (10)
C14	0.0243 (11)	0.0253 (14)	0.0234 (12)	0.0041 (10)	0.0040 (10)	0.0020 (10)
C13	0.0208 (11)	0.0247 (13)	0.0136 (10)	−0.0026 (9)	0.0053 (9)	0.0001 (9)
C16	0.0237 (11)	0.0287 (14)	0.0170 (11)	−0.0045 (10)	0.0017 (9)	−0.0036 (10)
C10	0.0303 (12)	0.0354 (16)	0.0360 (14)	−0.0049 (11)	0.0215 (12)	−0.0120 (12)

### Geometric parameters (Å, °)

**Table:**

S1—O2	1.4270 (15)	C17—C18	1.386 (3)
S1—O3	1.4285 (16)	C17—H17	0.9500
S1—N1	1.6339 (17)	C18—C13	1.395 (3)
S1—C1	1.760 (2)	C18—H18	0.9500
O5—C9	1.323 (3)	C11—C12	1.521 (3)
O5—C10	1.449 (3)	C11—H11A	0.9900
O1—C7	1.341 (3)	C11—H11B	0.9900
O1—H1	0.92 (3)	C3—C4	1.387 (3)
O4—C9	1.227 (3)	C3—H3	0.9500
N1—C8	1.429 (3)	C15—C14	1.379 (3)
N1—C11	1.485 (3)	C15—C16	1.384 (3)
C1—C2	1.387 (3)	C15—H15	0.9500
C1—C6	1.400 (3)	C4—H4	0.9500
C7—C8	1.361 (3)	C12—C13	1.511 (3)
C7—C6	1.475 (3)	C12—H12A	0.9900
C8—C9	1.459 (3)	C12—H12B	0.9900
C5—C4	1.382 (3)	C14—C13	1.387 (3)
C5—C6	1.392 (3)	C14—H14	0.9500
C5—H5	0.9500	C16—H16	0.9500
C2—C3	1.389 (3)	C10—H10A	0.9800
C2—H2	0.9500	C10—H10B	0.9800
C17—C16	1.380 (3)	C10—H10C	0.9800
			
O2—S1—O3	119.52 (9)	C13—C18—H18	119.9
O2—S1—N1	108.21 (9)	N1—C11—C12	114.02 (17)
O3—S1—N1	108.08 (9)	N1—C11—H11A	108.7
O2—S1—C1	107.13 (9)	C12—C11—H11A	108.7
O3—S1—C1	110.45 (10)	N1—C11—H11B	108.7
N1—S1—C1	102.04 (9)	C12—C11—H11B	108.7
C9—O5—C10	116.10 (18)	H11A—C11—H11B	107.6
C7—O1—H1	103.7 (17)	C4—C3—C2	120.5 (2)
C8—N1—C11	116.93 (16)	C4—C3—H3	119.7
C8—N1—S1	113.41 (13)	C2—C3—H3	119.7
C11—N1—S1	119.16 (14)	C14—C15—C16	120.3 (2)
C2—C1—C6	121.8 (2)	C14—C15—H15	119.9
C2—C1—S1	120.63 (17)	C16—C15—H15	119.9
C6—C1—S1	117.50 (16)	C5—C4—C3	120.9 (2)
O1—C7—C8	123.2 (2)	C5—C4—H4	119.6
O1—C7—C6	114.46 (19)	C3—C4—H4	119.6
C8—C7—C6	122.3 (2)	C13—C12—C11	111.30 (18)
C7—C8—N1	121.54 (19)	C13—C12—H12A	109.4
C7—C8—C9	120.0 (2)	C11—C12—H12A	109.4
N1—C8—C9	118.38 (19)	C13—C12—H12B	109.4
C4—C5—C6	119.7 (2)	C11—C12—H12B	109.4
C4—C5—H5	120.1	H12A—C12—H12B	108.0
C6—C5—H5	120.1	C15—C14—C13	121.0 (2)
O4—C9—O5	123.2 (2)	C15—C14—H14	119.5
O4—C9—C8	122.9 (2)	C13—C14—H14	119.5
O5—C9—C8	113.92 (19)	C14—C13—C18	118.5 (2)
C5—C6—C1	118.8 (2)	C14—C13—C12	121.3 (2)
C5—C6—C7	121.3 (2)	C18—C13—C12	120.1 (2)
C1—C6—C7	119.92 (19)	C17—C16—C15	119.4 (2)
C1—C2—C3	118.3 (2)	C17—C16—H16	120.3
C1—C2—H2	120.8	C15—C16—H16	120.3
C3—C2—H2	120.8	O5—C10—H10A	109.5
C16—C17—C18	120.5 (2)	O5—C10—H10B	109.5
C16—C17—H17	119.8	H10A—C10—H10B	109.5
C18—C17—H17	119.8	O5—C10—H10C	109.5
C17—C18—C13	120.3 (2)	H10A—C10—H10C	109.5
C17—C18—H18	119.9	H10B—C10—H10C	109.5
			
O2—S1—N1—C8	59.99 (16)	C4—C5—C6—C7	−178.7 (2)
O3—S1—N1—C8	−169.24 (14)	C2—C1—C6—C5	−1.3 (3)
C1—S1—N1—C8	−52.79 (16)	S1—C1—C6—C5	176.40 (16)
O2—S1—N1—C11	−156.23 (15)	C2—C1—C6—C7	178.87 (19)
O3—S1—N1—C11	−25.46 (18)	S1—C1—C6—C7	−3.5 (3)
C1—S1—N1—C11	90.99 (17)	O1—C7—C6—C5	−18.7 (3)
O2—S1—C1—C2	99.50 (18)	C8—C7—C6—C5	162.8 (2)
O3—S1—C1—C2	−32.19 (19)	O1—C7—C6—C1	161.14 (18)
N1—S1—C1—C2	−146.92 (17)	C8—C7—C6—C1	−17.4 (3)
O2—S1—C1—C6	−78.19 (17)	C6—C1—C2—C3	0.3 (3)
O3—S1—C1—C6	150.11 (15)	S1—C1—C2—C3	−177.31 (16)
N1—S1—C1—C6	35.39 (18)	C16—C17—C18—C13	−0.4 (3)
O1—C7—C8—N1	179.24 (18)	C8—N1—C11—C12	71.0 (2)
C6—C7—C8—N1	−2.4 (3)	S1—N1—C11—C12	−71.6 (2)
O1—C7—C8—C9	−3.3 (3)	C1—C2—C3—C4	0.5 (3)
C6—C7—C8—C9	175.06 (18)	C6—C5—C4—C3	−0.6 (3)
C11—N1—C8—C7	−103.1 (2)	C2—C3—C4—C5	−0.4 (3)
S1—N1—C8—C7	41.5 (2)	N1—C11—C12—C13	−168.72 (18)
C11—N1—C8—C9	79.4 (2)	C16—C15—C14—C13	−0.1 (3)
S1—N1—C8—C9	−136.00 (16)	C15—C14—C13—C18	0.5 (3)
C10—O5—C9—O4	−0.2 (3)	C15—C14—C13—C12	−179.6 (2)
C10—O5—C9—C8	179.15 (18)	C17—C18—C13—C14	−0.2 (3)
C7—C8—C9—O4	3.4 (3)	C17—C18—C13—C12	179.85 (19)
N1—C8—C9—O4	−179.04 (19)	C11—C12—C13—C14	−70.4 (3)
C7—C8—C9—O5	−175.93 (18)	C11—C12—C13—C18	109.5 (2)
N1—C8—C9—O5	1.6 (3)	C18—C17—C16—C15	0.8 (3)
C4—C5—C6—C1	1.4 (3)	C14—C15—C16—C17	−0.6 (3)

### Hydrogen-bond geometry (Å, °)

**Table:**

*D*—H···*A*	*D*—H	H···*A*	*D*···*A*	*D*—H···*A*
C2—H2···O3^i^	0.95	2.55	3.391 (3)	147
C17—H17···O2^ii^	0.95	2.53	3.205 (3)	128
C18—H18···O4^iii^	0.95	2.49	3.308 (3)	145
O1—H1···O4	0.92 (3)	1.74 (3)	2.583 (2)	152 (3)

Symmetry codes: (i) −*x*+1, −*y*, −*z*+1; (ii) *x*−1, *y*, *z*−1; (iii) *x*, −*y*+1/2, *z*−1/2.
